# Supplementary figures and images for: Non-lethal exposure to H2O2 boosts bacterial survival and evolvability against oxidative stress
Source: PLoS Genet. 2020 Mar 12;16(3):e1008649. doi: 10.1371/journal.pgen.1008649 (PMC7093028; doi:10.1371/journal.pgen.1008649)

**A**

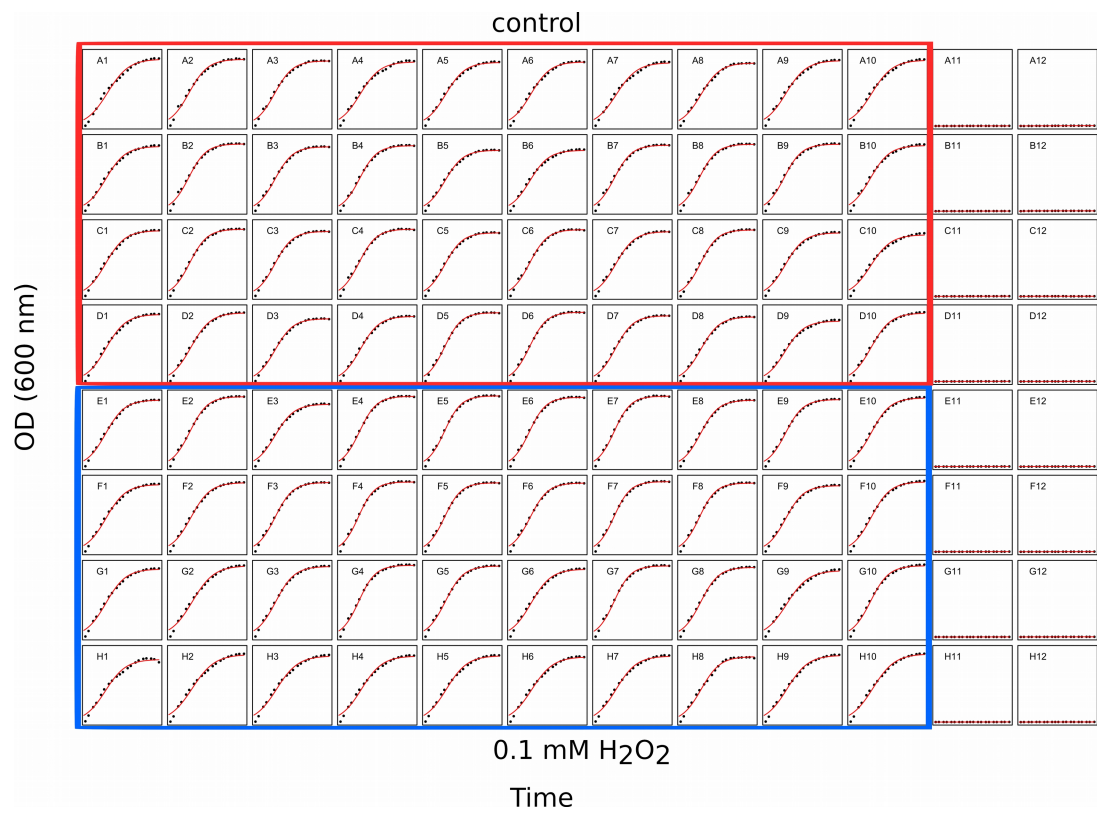

**B**

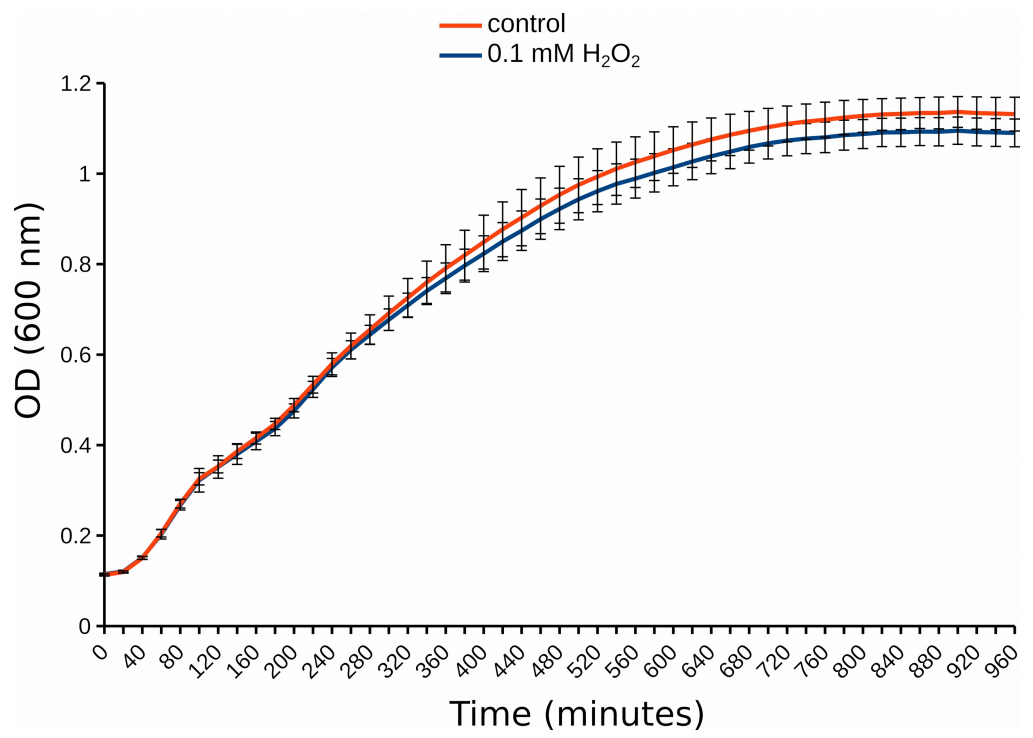

Supplement: S1 Fig — Panel A represents a graphical model fitting of individual curves plotted by Growthcurver R package [60]. Panel B shows the average growth curves from 40 independent replicas per each situation (0.1 mM H2O2 versus control). (PDF) [file pgen.1008649.s001.pdf]

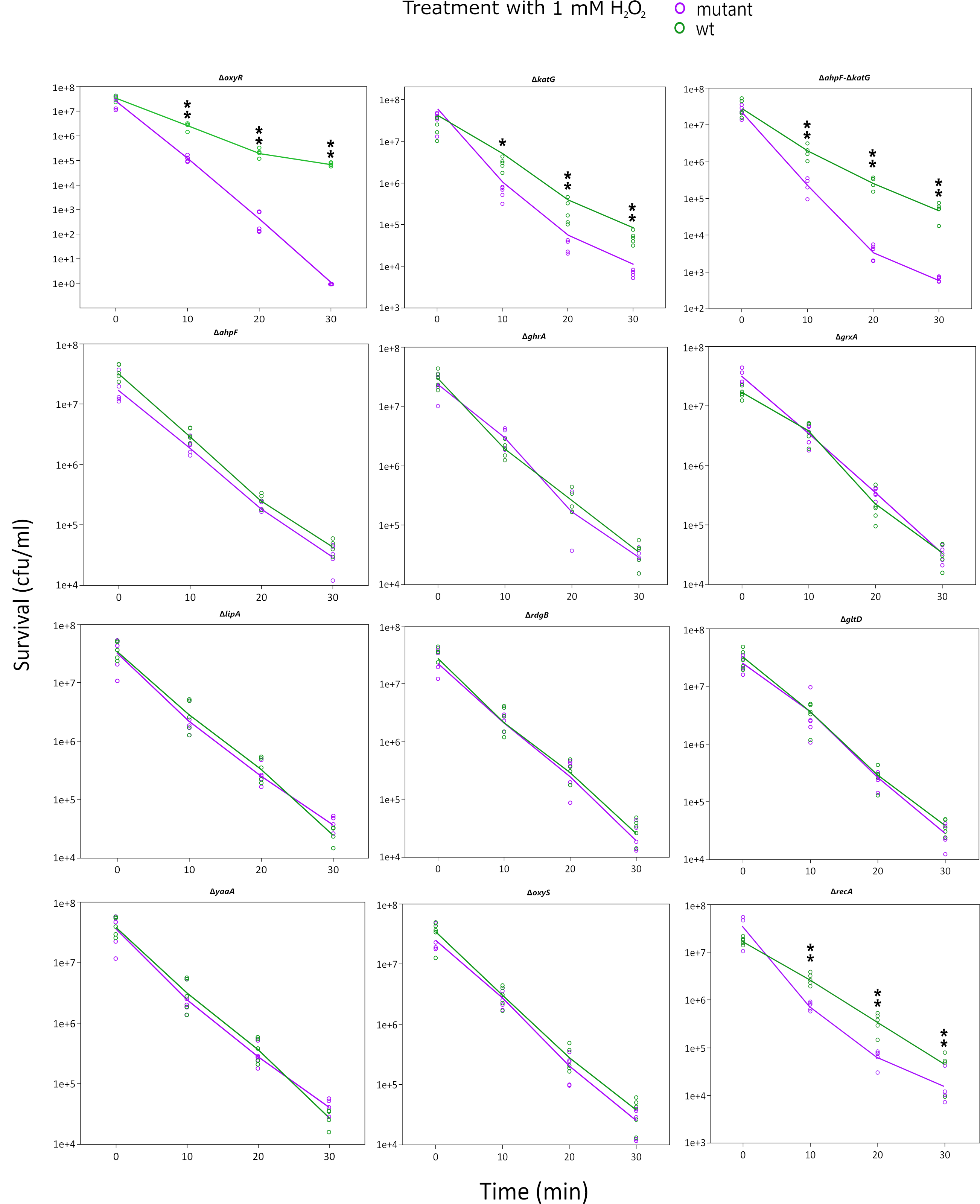

Supplement: S2 Fig — Asterisks represent significant differences between the wild-type (wt) strain and its derivatives mutants (Welch’s test, one asterisk for p<0.05 and two asterisks for p<0.01). (TIF) [file pgen.1008649.s002.tif]

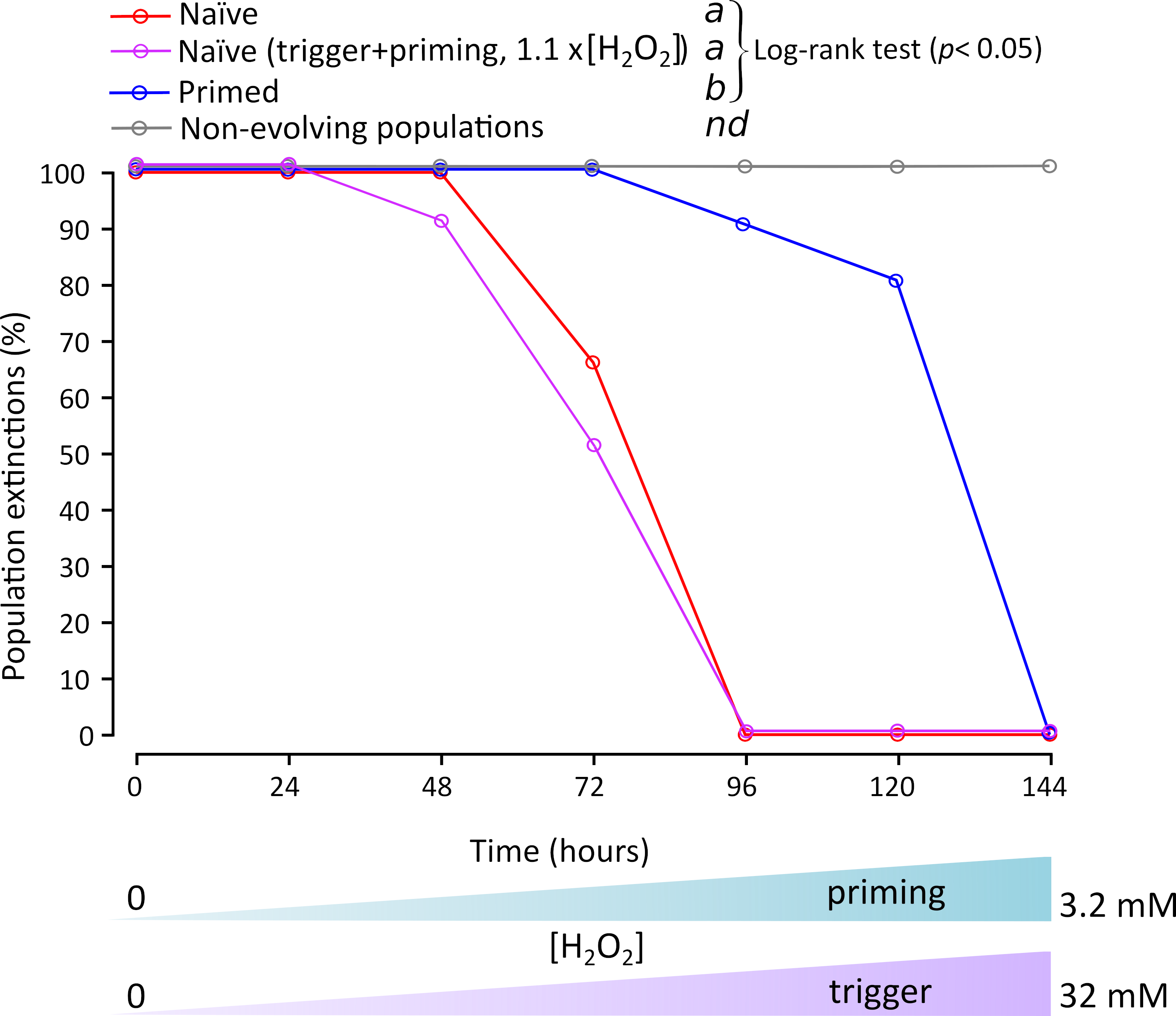

Supplement: S3 Fig — The extinction was perceived by negative growth in the next passage and by the absence of growth in LB plates during contamination controls. Non-evolving population control (grey line, 20 populations) is presented. Evolvability differs between the two naïve population groups (red and magenta lines) and primed populations (blue line). Equal letter represents no statistical differences while the same letter indicates significant differences in pair-wise comparison (Log-rank test, p< 0.05). Differences with non-evolved populations were not determined. (TIF) [file pgen.1008649.s003.tif]

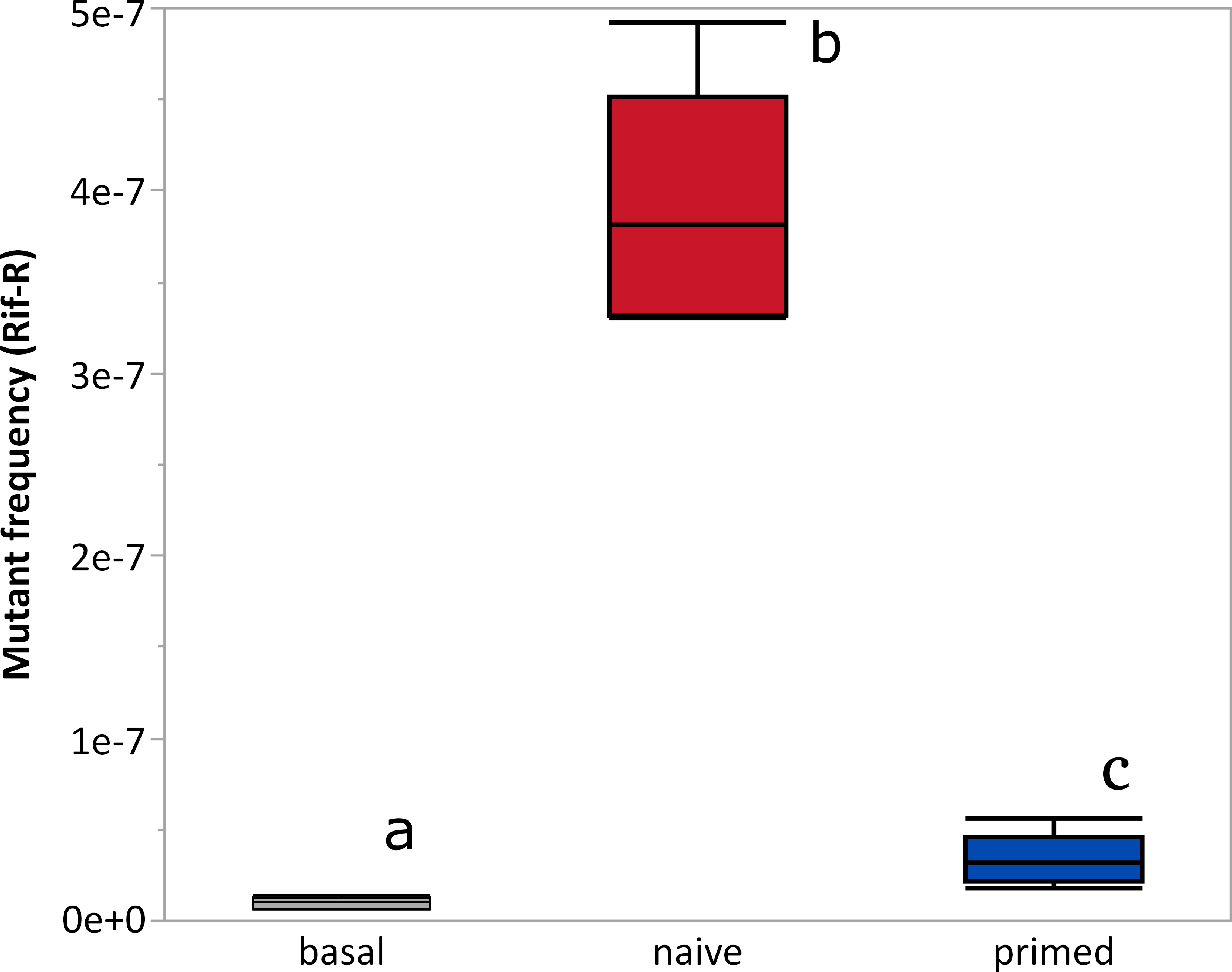

Supplement: S5 Fig — Naïve and primed cells (pre-treated with 0.1 mM, 30 minutes in advanced) cultures challenged with 1 mM, allowed to recover and plated in rifampicin (100 μg/ml). The basal level of mutagenesis for non-pre-treated, non-challenged cells is also shown. Every sample consisted of five independent replications. Letters denote significant differences (Welch’s test, p = 0.03 for basal level versus primed, p<0.01 for both basal versus naïve and primed versus naïve). (TIF) [file pgen.1008649.s005.tif]
