# Supplementary material for: Non-lethal exposure to H2O2 boosts bacterial survival and evolvability against oxidative stress
Source: PLoS Genet. 2020 Mar 12;16(3):e1008649. doi: 10.1371/journal.pgen.1008649 (PMC7093028; doi:10.1371/journal.pgen.1008649)
Supplement: S2 Table — H2O2 concentrations were determined for 0, 15 and 30 minutes after the addition of H2O2 using the Pierce Quantitative Peroxide kit (Thermo Scientific, Germany). The shown values represent the mean of the supernatant from three individual cultures and their standard deviations. (PDF) [file pgen.1008649.s007.pdf]

Table S2. Quantitative H_2O_2 determination of *E. coli* culture supernatants after a treatment with 1 mM after priming bacteria with 0.1 mM H_2O_2 in comparison to naïve cells. H_2O_2 concentrations were determined for 0, 15 and 30 minutes after the addition of H_2O_2 using the Pierce Quantitative Peroxide kit (Thermo Scientific, Germany). The shown values represent the mean of the supernatant from three individual cultures and their standard deviations.

	Treatment [H_2O_2] µM (*mean*± *sd*)		Statistical inferences	
Time (min)	Naive	Primed	Fold-change ratio (naive/primed)	*p*-value
0	1062.03±44.90	1053.97± 69.58	0.99	0.4380
15	723.68±62.43	140.46±33.45	5.15	0.00014
30	379.02±49.56	22.37 ±23.06	16.94	0.00035
