# Supplementary material for: Non-lethal exposure to H2O2 boosts bacterial survival and evolvability against oxidative stress
Source: PLoS Genet. 2020 Mar 12;16(3):e1008649. doi: 10.1371/journal.pgen.1008649 (PMC7093028; doi:10.1371/journal.pgen.1008649)
Supplement: S6 Table — (PDF) [file pgen.1008649.s011.pdf]

Table S6. Transcripts differentially expressed (±2 log2) in the fraction of small of RNA <200 nt)during the decay of hydrogen peroxide response.

Gene	Log2 fold change	P-value	Description
oxyS	3.73	8.24E-15	OxyS sRNA regulates genes in response to H2O2
rbsA	3.12	2.83E-09	Ribose import ATP-binding protein RbsA
rplW	3.10	8.37E-08	50S ribosomal protein L23
cyoB	2.76	1.23E-06	Cytochrome bo(3) ubiquinol oxidase subunit 1
rplB	2.45	4.73E-16	50S ribosomal protein L2
rpsJ	2.42	3.43E-10	30S ribosomal protein S10
rplC	2.24	5.83E-14	50S ribosomal protein L3
fruB	2.20	3.59E-04	Multiphosphoryl transfer protein
leuZ	2.11	1.15E-08	Leucine tRNA(GAG) 4
ptsG	2.10	3.73E-10	PTS system glucose-specific EIICB component
rpsC	2.08	4.01E-07	30S ribosomal protein S3
rplX	2.06	1.30E-05	50S ribosomal protein L24
rpsE	2.03	2.05E-08	30S ribosomal protein S5
raiA	-2.01	5.78E-12	Ribosome-associated inhibitor A
dcuA	-2.05	1.51E-03	Anaerobic C4-dicarboxylate transporter DcuA
deoA	-2.11	1.43E-04	Thymidine phosphorylase
adhE	-2.20	1.15E-06	Aldehyde-alcohol dehydrogenase
gatD	-2.22	1.41E-03	Galactitol-1-phosphate 5-dehydrogenase
dnaK	-2.56	7.42E-05	Chaperone protein DnaK
yhjX	-2.60	1.19E-04	Uncharacterized MFS-type transporter YhjX
glnA	-2.62	1.12E-04	Glutamine synthetase
treB	-2.66	5.70E-07	PTS system trehalose-specific EIIBC component
glpT	-2.67	1.24E-06	Glycerol-3-phosphate transporter
clpA	-2.76	1.36E-05	ATP-dependent Clp protease ATP-binding subunit ClpA
glpA	-2.78	6.94E-05	Anaerobic glycerol-3-phosphate dehydrogenase subunit A
nanA	-2.80	6.12E-05	N-acetylneuraminate lyase
gatC	-2.88	2.15E-09	NA
glpQ	-2.98	6.54E-09	Glycerophosphoryl diester phosphodiesterase
glpK	-2.99	1.79E-08	Glycerol kinase
glpB	-3.02	1.28E-05	Anaerobic glycerol-3-phosphate dehydrogenase subunit B
glpD	-3.06	6.99E-09	Aerobic glycerol-3-phosphate dehydrogenase
dtpB	-3.08	2.06E-06	Dipeptide and tripeptide permease B
rbbA	-3.26	1.73E-06	Ribosome-associated ATPase
malM	-3.61	5.57E-08	Maltose operon periplasmic protein
tnaA	-3.78	9.07E-11	Tryptophanase
yjiY	-4.20	4.75E-15	Inner membrane protein YjiY
