# Supplementary material for: Non-lethal exposure to H2O2 boosts bacterial survival and evolvability against oxidative stress
Source: PLoS Genet. 2020 Mar 12;16(3):e1008649. doi: 10.1371/journal.pgen.1008649 (PMC7093028; doi:10.1371/journal.pgen.1008649)
Supplement: S7 Table — (PDF) [file pgen.1008649.s012.pdf]

Table S7. Transcripts differentially expressed (± 2.5 log2) in the fraction of large RNA (>200 nt) during the decay of hydrogen peroxide response.

Gene	Log2 fold change	P-value	Description
oxyS	4.02	2.34E-10	OxyS sRNA regulates genes in response to H2O2
ptsG	3.92	4.87E-96	PTS system glucose-specific EIICB component
rbsA	3.90	3.78E-73	Ribose import ATP-binding protein RbsA
fruB	3.66	2.13E-105	Multiphosphoryl transfer protein
rplV	3.48	7.18E-139	50S ribosomal protein L22
rpsQ	3.47	5.52E-110	30S ribosomal protein S17
yihL	3.42	2.45E-10	Uncharacterized HTH-type transcriptional regulator YihL
rpsC	3.26	4.84E-164	30S ribosomal protein S3
rpsS	3.22	1.96E-154	30S ribosomal protein S19
rplP	3.21	9.71E-115	50S ribosomal protein L16
rplW	3.13	5.30E-173	50S ribosomal protein L23
rpmC	3.10	3.41E-40	50S ribosomal protein L29
cyoD	3.05	6.09E-19	Cytochrome bo(3) ubiquinol oxidase subunit 4
rplB	2.99	2.76E-161	50S ribosomal protein L2
cyoC	2.98	3.55E-32	Cytochrome bo(3) ubiquinol oxidase subunit 3
rpsA	2.74	1.13E-167	30S ribosomal protein S1
rplO	2.71	1.56E-73	50S ribosomal protein L15
rplQ	2.71	4.27E-113	50S ribosomal protein L17
rplD	2.69	1.14E-102	50S ribosomal protein L4
sdaB	2.66	4.27E-29	L-serine dehydratase 2
fecB	2.62	2.78E-06	Fe(3+) dicitrate-binding periplasmic protein
fruK	2.62	1.21E-36	1-phosphofructokinase
rpmD	2.61	1.67E-33	50S ribosomal protein L30
cyoB	2.60	2.70E-42	Cytochrome bo(3) ubiquinol oxidase subunit 1
rbsD	2.59	2.25E-95	D-ribose pyranase
secY	2.53	1.04E-157	Protein translocase subunit SecY
rpsE	2.52	9.45E-50	30S ribosomal protein S5
rplC	2.51	6.52E-149	50S ribosomal protein L3
yicG	-2.53	3.42E-10	UPF0126 inner membrane protein YicG
glgX	-2.55	1.50E-15	Glycogen debranching enzyme
yhaM	-2.55	2.25E-07	NA
treA	-2.56	4.75E-07	Periplasmic trehalase
htpG	-2.56	2.54E-32	Chaperone protein HtpG
frdB	-2.58	4.04E-04	Fumarate reductase iron-sulfur subunit
ftnB	-2.59	3.02E-05	Bacterial non-heme ferritin-like protein
aceA	-2.60	1.93E-13	Isocitrate lyase
srlE	-2.61	1.06E-07	Glucitol/sorbitol-specific phosphotransferase enzyme IIB component
glnA	-2.61	3.74E-60	Glutamine synthetase
ygfF	-2.65	2.67E-04	Uncharacterized oxidoreductase YgfF
nanE	-2.67	1.78E-05	Putative N-acetylmannosamine-6-phosphate 2-epimerase
malM	-2.68	6.31E-15	Maltose operon periplasmic protein
flgG	-2.71	6.60E-04	Flagellar basal-body rod protein FlgG
glpD	-2.74	8.49E-13	Aerobic glycerol-3-phosphate dehydrogenase
tnaC	-2.75	2.86E-25	Tryptophanase leader peptide
yhhJ	-2.75	1.31E-08	Inner membrane transport permease YhhJ
glnQ	-2.75	1.71E-09	Glutamine transport ATP-binding protein GlnQ
rbbA	-2.76	3.25E-10	Ribosome-associated ATPase
glnL	-2.78	2.37E-09	Nitrogen regulation protein NR(II)
tdcE	-2.78	7.91E-09	PFL-like enzyme TdcE

Gene	Log2 fold change	P-value	Description
glnH	-2.80	4.66E-40	Glutamine-binding periplasmic protein
nanT	-2.82	6.98E-08	Putative sialic acid transporter
dppF	-2.86	1.36E-05	Dipeptide transport ATP-binding protein DppF
gatD	-2.88	2.42E-05	Galactitol-1-phosphate 5-dehydrogenase
malG	-2.91	1.58E-06	Maltose transport system permease protein MalG
srlD	-3.00	2.31E-16	Sorbitol-6-phosphate 2-dehydrogenase
preT	-3.01	2.93E-13	NAD-dependent dihydropyrimidine dehydrogenase subunit PreT
glnP	-3.02	1.34E-11	Glutamine transport system permease protein GlnP
hybA	-3.06	5.68E-08	Hydrogenase-2 operon protein HybA
dppD	-3.08	6.15E-05	Dipeptide transport ATP-binding protein DppD
glgA	-3.09	2.36E-18	Glycogen synthase
preA	-3.11	9.50E-18	NAD-dependent dihydropyrimidine dehydrogenase subunit PreA
nanA	-3.18	7.54E-53	N-acetylneuraminate lyase
glgP	-3.25	8.54E-28	Glycogen phosphorylase
nrdD	-3.27	3.28E-07	Anaerobic ribonucleoside-triphosphate reductase
glgC	-3.32	5.62E-28	Glucose-1-phosphate adenylyltransferase
nanK	-3.47	4.04E-10	N-acetylmannosamine kinase
glpK	-3.47	7.37E-98	Glycerol kinase
srlB	-3.53	1.96E-05	Glucitol/sorbitol-specific phosphotransferase enzyme IIA component
lldD	-3.60	1.00E-09	L-lactate dehydrogenase
yhcH	-3.85	2.78E-10	Uncharacterized protein YhcH
yjiY	-3.87	2.85E-24	Inner membrane protein YjiY
dsdX	-3.88	1.18E-11	DsdX permease
clpB	-3.91	9.50E-71	Chaperone protein ClpB
glpQ	-4.05	1.21E-59	Glycerophosphoryl diester phosphodiesterase
yhjX	-4.09	3.34E-44	Uncharacterized MFS-type transporter YhjX
tdcD	-4.44	7.60E-07	Propionate kinase
cadB	-4.67	1.67E-09	Probable cadaverine/lysine antiporter
tnaA	-4.80	8.72E-29	Tryptophanase
glpB	-4.94	9.23E-20	Anaerobic glycerol-3-phosphate dehydrogenase subunit B
tnaB	-4.99	3.17E-13	Low affinity tryptophan permease
dsdA	-5.06	3.05E-36	D-serine dehydratase
cadA	-5.34	8.06E-13	Lysine decarboxylase. inducible
glpC	-5.97	1.59E-16	Anaerobic glycerol-3-phosphate dehydrogenase subunit C
