# Supplementary material for: Non-lethal exposure to H2O2 boosts bacterial survival and evolvability against oxidative stress
Source: PLoS Genet. 2020 Mar 12;16(3):e1008649. doi: 10.1371/journal.pgen.1008649 (PMC7093028; doi:10.1371/journal.pgen.1008649)
Supplement: S8 Table — (PDF) [file pgen.1008649.s013.pdf]

Gene	Gene function	Type	Result	Expression	Std. Error	95% C.I.	P(H1)
asnA	Asparagine synthase A	REF		2.3			
metL	Bifunctional aspartokinase/homoserine dehydrogenase 2	REF		-2.3			
mntH	Mn transport	TRG	UP	55.5	51.567 - 59.843	49.843 - 61.254	0.000
sufA	Scaffold protein for assembly of iron-sulfur clusters; facilitates delivery to target proteins; Fe-S transfer protein	TRG	UP	18.2	14.303 - 21.615	13.185 - 25.838	0.000
fur	Ferric uptake regulation	TRG	NDE	-1.1	0.845 - 0.942	0.832 - 0.959	0.090
ahpC	Alkyl hydroperoxide reductase, subunit C; reduced by the AhpF subunit. protects aerobic, phosphate-starved cells from oxidative damage	TRG	UP	7.3	6.616 - 8.502	6.507 - 8.808	0.000
yaaA	Peroxide resistance protein, lowers intracellular iron	TRG	UP	2.2	1.954 - 2.526	1.862 - 2.610	0.000
oxyR	Oxidative and nitrosative stress transcriptional regulator	TRG	NDE	-4.9	0.185 - 0.222	0.179 - 0.238	0.079
katG	Catalase-peroxidase HPI, heme b-containing; hydroperoxidase I. KatG protects aerobic, phosphate-starved cells from oxidative damage	TRG	UP	25.7	19.066 - 33.678	17.159 - 42.580	0.011
ahpF	Alkyl hydroperoxide reductase, subunit F; NAD(P)H:peroxiredoxin oxidoreductase, reduces AhpC; contains one FAD/monomer	TRG	UP	5.6	4.739 - 6.291	4.489 - 7.307	0.011
dps	Stress-induced Fe-binding and storage protein; forms biocrystals with DNA; copper homeostatsis. Dps confers starvation-induced resistance to H2O2.	TRG	UP	3.1	2.676 - 3.484	2.530 - 3.915	0.021
