# Supplementary material for: Non-lethal exposure to H2O2 boosts bacterial survival and evolvability against oxidative stress
Source: PLoS Genet. 2020 Mar 12;16(3):e1008649. doi: 10.1371/journal.pgen.1008649 (PMC7093028; doi:10.1371/journal.pgen.1008649)
Supplement: S9 Table — (PDF) [file pgen.1008649.s014.pdf]

Table S9. Strains and plasmids used in this work and their relevant phenotypes

Strain	Relevant phenotype	Reference
E. coli MG1655 WT	F⁻ λ⁻	Lab stock
E. coli MG1655 oxyR::scar	*F⁻ λ⁻ oxyR::scar*	this work
E. coli MG1655 katG::scar	*F⁻ λ⁻ katG::scar*	this work
E. coli MG1655 ahpF::scar	*F⁻ λ⁻ ahpF::scar*	this work
E. coli MG1655 ahpF::scar katG::scar	*F⁻ λ⁻ ahpF::scar katG::scar*	this work
E. coli MG1655 grxA::scar	*F⁻ λ⁻ grxA::scar*	this work
E. coli MG1655 ghrA::scar	*F⁻ λ⁻ ghrA::scar*	this work
E. coli MG1655 lipA::scar	*F⁻ λ⁻ lipA::scar*	this work
E. coli MG1655 rdgB::scar	*F⁻ λ⁻ rdgB::scar*	this work
E. coli MG1655 yaaA::scar	*F⁻ λ⁻ yaaA::scar*	this work
E. coli MG1655 recA::scar	*F⁻ λ⁻ recA::scar*	this work
E. coli MG1655 fimE (Δ1 bp, 248 nt)	*fimE* Δ1 bp, position 248	this work
E. coli MG1655 insB1→flhD Δ1 bp	Δ1 bp, position 1978504	this work
E. coli MG1655 insB1→flhD Δ10 bp	Δ10 bp, position 1978493	this work
E. coli MG1655 oxyS::Cm	*F⁻ λ⁻ oxyS::Cm*	27
E. coli BW25113 oxyR::Kan	*oxyR::Kan*	57
E. coli BW25113 katG::Kan	*katG::Kan*	57
E. coli BW25113 ahpF::Kan	*ahpF::Kan*	57
E. coli BW25113 grxA::Kan	*grxA::Kan*	57
E. coli BW25113 ghrA::Kan	*ghrA::Kan*	57
E. coli BW25113 lipA::Kan	*lipA::Kan*	57
E. coli BW25113 rdgB::Kan	*rdgB::Kan*	57
E. coli BW25113 yaaA::Kan	*yaaA::Kan*	57
E. coli BW25113 recA::Kan	*recA::Kan*	57
pCA24N	Cloning vector, Cm-R	36
pCA24N-*fimE*	pCA24N carrying *fimE*, Cm-R	36
pBAD24	Cloning vector, Amp-R	80
pVN15	pBAD24 carrying *flhDC* operon, Amp-R	80
