# Supplementary material for: Non-lethal exposure to H2O2 boosts bacterial survival and evolvability against oxidative stress
Source: PLoS Genet. 2020 Mar 12;16(3):e1008649. doi: 10.1371/journal.pgen.1008649 (PMC7093028; doi:10.1371/journal.pgen.1008649)
Supplement: S10 Table — (PDF) [file pgen.1008649.s015.pdf]

Table S10. Primers used for amplification gene mutants from the Keio collection which PCR products were used to transfer the mutations to the *E. coli* MG1655.

Gene	Forward primer (5'--->3')	Reverse primer (5'--->3')
oxyR	ACTCTCGAAACGGGCAGTG	GGTCAGGCGATTATGGAACAG
katG	GATCTCAACTATCGCATCCGTG	CACAACCAGGCCACTGAT
ahpF	ACCTAATTCTTCGGGTGCTG	GGTGCTGGTTGTGCGTTAA
ghrA	CACCGACCACGGATTTGTTATG	GCATCTTCCATATCCGGGCC
grxA	CCTCTGCAAAGTGAGCCTTC	CACCCTGTTCGATGCTCATTAT
lipA	ACCGCTTTGGCTGCTTTC	CATAAAGAGTGACGTGGCGA
rdgB	CTGGTGAAGTTTCTCGGTAAGC	CGTACCACCGCCAATAAAGA
gltD	GCGCGGTGAAGAGATTCTG	GGAAAGGTCAAACGCTCATGC
yaaA	CCCGGTGTTTGATCCATTGC	CAGGCAAACATCACCGCT
recA	GTCGTCAGGCTACTGCGTATG	GTCGCAGTTCTTGCTCACTG
