# Supplementary material for: Non-lethal exposure to H2O2 boosts bacterial survival and evolvability against oxidative stress
Source: PLoS Genet. 2020 Mar 12;16(3):e1008649. doi: 10.1371/journal.pgen.1008649 (PMC7093028; doi:10.1371/journal.pgen.1008649)
Supplement: S11 Table — (PDF) [file pgen.1008649.s016.pdf]

Table S11. Primers used for relative gene expression quantification by real time PCR (qPCR). RNA was extracted after a treatment of *E. coli* MG1655 with H_2O_2 0.1 mM during 30 minutes.

Gene	EcoGene ID	Forward primer (5'--->3')	Reverse primer (5'--->3')
ahpC	EG11384	GCAGCACAGTACGTAGCTTCTCA	GCCAGAGTTGCTTCACCTTCTT
ahpF	EG11385	CTGAACCTGATGAGCGTA	GACCAAACTCTTTCCCGT
dps	EG11415	GTACATGAAATGCTGGATGG	TGCTGTTGATAACTTGAGTG
fur	EG10359	CAGCAACATCACCACGATCAC	TGGAATCATCACTAAATTCGATAACC
katG	EG10511	CAAATGCCCGTTCCATC	TTAACAGGTCAACACGAAGT
mntH	EG14157	GTTATTCCGCCACCAAATG	TAGCCATCATCGCCAGA
oxyR	EG10681	GCCAGCCGACGCTTAGC	AACATCACGCCCAGCTCATC
sufA	EG11378	TTGATGGCACGGAAGTC	TGGGCTTTAGGGTTGTG
yaaA	EG10011	GAAACCTTCAGCGAAGACGATT	CCATACAAGCCGGAAAGCA
metL	EG10590	TGAGCAGGATGAAGAGTCGTTG	CCGTGGCTGGCGAAATCAAG
asnA	EG10091	CTGGGCGGGAATTAAAGCAACC	CTCCTGGCTGTGTACGAAGTGG
